# Real World Management of Cytopenias and Infections in Patients With Myelofibrosis Treated With Ruxolitinib

**DOI:** 10.1002/jha2.70007

**Published:** 2025-03-21

**Authors:** Liesl A. Butler, Cecily Forsyth, Claire Harrison, Andrew C. Perkins

**Affiliations:** ^1^ Monash University Melbourne Victoria Australia; ^2^ Alfred Health Melbourne Victoria Australia; ^3^ Central Coast Haematology North Gosford New South Wales Australia; ^4^ Guy's and St Thomas' NHS Foundation Trust London UK

## Abstract

**Introduction:**

Ruxolitinib was the first JAK2 inhibitor approved for the treatment of primary and secondary myelofibrosis. It is currently used worldwide as first‐line therapy for advanced disease (intermediate‐2 and high‐risk) and is effective in polycythaemia vera (PV) and essential thrombocythaemia (ET), but not funded for this indication in many countries. Ruxolitinib has proven benefits with respect to symptom control, reduction in spleen size and prolongation of survival; however, it rarely induces a substantial reduction in allele burden and never provides a cure. Moreover, there are frequently encountered adverse effects and dosing issues that require careful management to optimise its therapeutic benefit.

**Methods and Results:**

In this case‐based review, we use seven informative common clinical scenarios to discuss appropriate investigation and management of cytopenias and infection issues.

**Conclusions:**

We make recommendations based on 15 years of experience in using ruxolitinib and other JAK inhibitors for the treatment of myelofibrosis. We discuss when allogeneic haematopoietic stem cell transplantation (AHSCT) should be considered and some of the currently available alternative JAK inhibitors and trial options when AHSCT is not an option.

## 1 Introduction

The management of myelofibrosis (MF) is guided by clinical or combined clinical and genetic scoring systems, which risk‐stratify patients to determine prognosis and subsequent treatment options [1, 2, 3, 4, 5]. Patients are grouped as either lower‐ or higher‐risk disease; patients with higher‐risk disease should be considered for AHSCT if fit; otherwise, symptom‐directed therapy is indicated. Current treatments include hydroxycarbamide, pegylated interferon, JAK inhibitors and anaemia‐directed drugs. Ruxolitinib was the first JAK inhibitor to be approved for use in intermediate and high‐risk MF and remains the standard of care today. It has been shown to be effective in decreasing spleen volume and alleviating symptoms, including in the hydroxycarbamide‐refractory setting [6, 7, 8]. Additionally, it can offer a long‐term survival benefit when compared with conventional therapies in higher‐risk patients [9]. However, the treatment course is frequently complicated by cytopenias, and the drug has significant immunosuppressive activity, so it can increase the risk of opportunistic infection and potentially secondary malignancies [6, 10, 11]. Furthermore, MF invariably progresses and often becomes less responsive or refractory to ruxolitinib therapy after a few years, when alternative treatments need to be pursued [12]. Nevertheless, ruxolitinib remains one of the most effective and readily available treatments for MF, and it is important to continue therapy at the most effective dose when possible. Herein, we outline several common clinical scenarios encountered in patients on ruxolitinib and detail strategies to maintain effective JAK inhibition and optimise therapeutic response.

## 2 Cytopenias

### 2.1 Anaemia

#### 2.1.1 Case Study 1

A 67‐year‐old female was diagnosed with JAK2‐positive ET (Table 1). Four years later, she developed progressive anaemia requiring transfusion (haemoglobin 76 g/L), splenomegaly (10 cm below the costal margin) and constitutional symptoms. A bone marrow biopsy confirmed evolution to post‐ET MF. The Dynamic International Prognostic Scoring System (DIPSS) risk score was intermediate‐2 with unremarkable cytogenetics/FISH, and next‐generation sequencing (NGS) demonstrated a JAK2 V617F mutation only (86% variant allele frequency [VAF]). She commenced ruxolitinib 10 mg BD in combination with the BCL‐2 inhibitor, navitoclax, as part of the REFINE study (NCT03222609) (platelets of 203 × 10^9^/L at initiation) in an effort to modify the natural history of the disease [13]. The symptoms and splenomegaly improved (the spleen reduced from 12 to 1 cm, and there was less fatigue); however, the patient required multiple navitoclax dose reductions due to cytopenias. The ruxolitinib dose was able to be maintained with regular 3‐weekly red blood cell (RBC) transfusions. She developed significant iron overload (ferritin 2092 mcg/L), and iron chelation with deferasirox was initiated. The patient remained on trial for almost a year but elected to change to an alternative anaemia‐specific study in an attempt to reduce her transfusion requirements. She received luspatercept/placebo in the INDEPENDENCE trial (NCT04717414), and her RBC transfusion requirements reduced by greater than 50% over 24 weeks, enabling her to continue on the blinded extension phase [14]. After almost 2 years of treatment, she progressed with 13% blasts on peripheral blood and was unable to remain on study. She continues on 10 mg BD ruxolitinib with RBC transfusions with no progression to blast phase (BP).

**TABLE 1:** Summary of clinical scenarios presented.

Issue	Case	Summary
Cytopenias		
Anaemia	1	67‐year‐old female, JAK2‐positive post‐ET MF with anaemia, splenomegaly and constitutional symptoms Ruxolitinib and navitoclax via REFINE (NCT03222609) Symptom and spleen improvement Navitoclax dose reductions due to cytopenias Transfusion‐dependent anaemia with iron overload requiring chelation Ruxolitinib plus luspatercept/placebo via INDEPENDENCE (NCT04717414) Reduced transfusion requirements Progression to accelerated phase so discontinued study
Thrombocytopenia	2	72‐year‐old male, JAK2‐positive symptomatic PMF with splenomegaly Ruxolitinib monotherapy Thrombocytopenia requiring dose reductions Poor spleen and symptom response Pacritinib versus best available therapy via PACIFICA (NCT03165734)
Infection		
Herpes zoster	3	60‐year‐old female, JAK2‐positive PMF with splenomegaly Hydroxycarbamide Constitutional symptoms and presence of peripheral blood blasts Ruxolitinib and navitoclax via REFINE (NCT03222609) Cytopenias requiring navitoclax dose reductions Improvement in symptoms and spleen size Herpes zoster managed with therapeutic dose then prophylactic antiviral therapy
Secondary malignancy		
Non‐melanomatous skin cancer	4	74‐year‐old male, JAK2‐positive post‐ET MF with splenomegaly and constitutional symptoms Ruxolitinib monotherapy Some spleen and symptom response Siramadlin plus ruxolitinib via ADORE (NCT04097821) Multiple SCCs and BCCs Cytopenias and lack of splenic/symptom improvement Ruxolitinib and navitoclax via REFINE (NCT03222609) Reduced spleen volume Metastatic Merkel cell carcinoma so unable to continue on trial Ruxolitinib monotherapy
Progressive disease		
Worsening MF and ruxolitinib response/refractoriness	5	61‐year‐old male, JAK2‐positive symptomatic PMF Ruxolitinib monotherapy Anaemia Navtemadlin via BOREAS (NCT03662126) Poor response Liver involvement of myelofibrosis Ruxolitinib and hydroxycarbamide Decrease in symptoms and spleen size Transfusion‐dependent anaemia Fedratinib via FREEDOM‐2 (NCT03952039) Improvement in symptoms, splenomegaly and transfusion‐dependence
	6	74‐year‐old male, JAK2‐positive post‐ET MF with splenomegaly Ruxolitinib monotherapy Spleen response but transfusion‐dependent anaemia Subsequent loss of response (splenomegaly, symptoms and leucocytosis) Ruxolitinib and navitoclax via REFINE (NCT03222609) Progressive splenomegaly and leucocytosis Ruxolitinib and hydroxycarbamide Decreased spleen size and symptoms Leucocytosis and thrombocytopenia Died
Accelerated and blast phase	7	72‐year‐old male, JAK2‐positive PMF with splenomegaly and constitutional symptoms Ruxolitinib monotherapy Worsening splenomegaly, symptoms and leucocytosis Rineterkib plus ruxolitinib via ADORE (NCT04097821) No change to disease status Retinopathy requiring withdrawal from study Ruxolitinib and navitoclax via TRANSFORM‐2 (NCT04468984) Progressive leucocytosis and thrombocytopenia Ruxolitinib and hydroxycarbamide Transformation to blast phase Azacitidine and venetoclax Pancytopenia, splenomegaly and infectious complications Supportive care and splenic radiotherapy Died

Abbreviations: BCCs, basal cell carcinomas; ET, essential thrombocytosis; PMF, primary myelofibrosis; SCCs, squamous cell carcinomas.

Anaemia in MF is frequently multifactorial; it may be disease‐related, secondary to therapy and/or due to unrelated pathology, such as gastrointestinal bleeding, haemolysis or nutritional deficiencies. A repeat bone marrow biopsy with cytogenetic and molecular testing can assist in determining whether disease progression (including the development of dysplasia) is contributing to progressive anaemia. Timing also provides insight into the aetiology, as ruxolitinib‐induced anaemia is dose‐dependent and frequently occurs in the early months of treatment [15]. The haemoglobin tends to reach a nadir (15 to 20 g/L from baseline) after approximately 12 weeks of therapy and usually improves (by 10 g/L) by Week 24 as the spleen shrinks [7]. Importantly, baseline anaemia is not a contraindication to the initiation of ruxolitinib with numerous analyses illustrating improvement in symptoms, spleen volume and survival in those with pre‐existing anaemia and transfusion dependence [8, 9, 16, 17]. The REALISE study (NCT02966353) used a novel ruxolitinib dosing strategy, demonstrating that the drug was still efficacious and safe in those with MF and anaemia when commenced at the reduced dose (10 mg BD) and gradually up‐titrated (to a maximum of 25 mg BD) [18].

In managing patients with anaemia on ruxolitinib, it is essential that regular blood monitoring is undertaken and appropriate dosing guidelines are followed. RBC transfusions should be administered when clinically indicated, with dose reductions to treatment as necessary. If a patient is well‐controlled on a particular dose of ruxolitinib, then management with transfusion is preferred given that a lower dose of ruxolitinib is less likely to control symptoms and splenomegaly; discontinuation should be avoided. A balance must be achieved between maintaining quality of life and avoiding iron overload when using red cells in the transfusion‐dependent population.

Iron chelation may need to be initiated to reduce the likelihood of developing complications related to iron overload and is generally considered when serum ferritin exceeds 1000 µg/L in chronically transfused individuals. Chelation therapy has demonstrated improvements in overall survival and haematological parameters in patients with lower‐risk myelodysplastic neoplasms, yet there is limited data surrounding the safety and efficacy of iron chelation in MF [19, 20, 21]. A relatively recent retrospective study of 69 patients with MF on ruxolitinib and deferasirox demonstrated that 48% of the cohort achieved a ferritin level less than 1000 µg/L or a reduction of 50% or more in ferritin level for 3 months or more, and only nine patients discontinued due to deferasirox‐related adverse events (with no unexpected toxicities reported) [22].

Additional therapies that may be considered alone or in combination with ruxolitinib for MF‐related anaemia include erythropoietin‐stimulating agents (ESAs), danazol and erythroid maturation agents. ESAs may be considered in eligible patients with a serum erythropoietin (EPO) less than 500 mU/mL; however, these agents may not be reimbursed for MF‐associated anaemia in some countries [23]. It is important to note that EPO measurements can be unreliable in the context of ruxolitinib therapy, so ideally, they should be performed prior to treatment initiation or following cessation. ESAs have shown variable responses, with one study of 163 patients reporting an anaemia response in up to 53% of individuals with a median response duration of 19 months [24]. Patients with fewer RBC transfusion requirements and lower baseline serum EPO levels are more likely to benefit from treatment, though responses are not predictable, and ultimately, patients become refractory to ESAs [25]. Improvement in anaemia (including in transfusion‐dependent patients) has also been observed with the use of synthetic androgen and danazol [26, 27]. Therapies targeting the transforming growth factor‐β (TGF‐β) superfamily, which are negative regulators of erythroid progenitor differentiation, are currently being investigated in the trial setting and are showing promise in ameliorating MF‐associated anaemia. Luspatercept, a Smad2/3‐pathway ligand trap that augments late‐stage erythropoiesis, has been evaluated in Phase 2 clinical trials, and the Phase 3 INDEPENDENCE study (NCT04717414) is now underway. In those with transfusion independence (in ACE‐536‐MF‐001 [NCT03194542]), 10% of patients on luspatercept alone and 21% of those on combination therapy with ruxolitinib achieved an anaemia response [28]. Whilst in the transfusion‐dependent population, transfusion independence was achieved in 10% and 27% of patients on single‐agent luspatercept or combined luspatercept/ruxolitinib, respectively [29]. Other inhibitors of TGF‐β signalling include sotatercept, which has shown safety and efficacy against anaemia in non‐transfusion and transfusion‐dependent patients, and elritercept, which is being tested in a Phase 2 study [30, 31]. Small molecule inhibitors and monoclonal antibodies that reduce hepcidin production are also in varying stages of development [32].

Momelotinib is an alternative JAK inhibitor (targeting JAK1/2) that has been associated with a lower incidence of anaemia and reduced transfusion requirements when compared to ruxolitinib (in both ruxolitinib‐naïve and ‐exposed patient populations) as part of the SIMPLIFY studies (NCT01969838, NCT02101268) [33, 34, 35]. It has activity against activin A receptor type 1 (ACVR1) and thus can improve erythropoiesis [36]. The recent MOMENTUM trial (NCT04173494), comparing the drug to danazol in patients with symptomatic MF and anaemia, has demonstrated a transfusion independence rate of 31% after 24 weeks of treatment [27, 37]. Where available, momelotinib should be considered as a first‐line option in treatment‐naïve patients with anaemia and splenomegaly.

Anaemia response in MF is generally defined by the International Working Group European LeukemiaNet criteria and recently a proposal has been put forth in an effort to harmonise the framework for defining anaemia severity and response to treatment [38, 39].

### 2.2 Thrombocytopenia

#### 2.2.1 Case Study 2

A 72‐year‐old male was referred for consideration of clinical trial participation for refractory MF (Table 1). He had JAK2‐positive disease that was diagnosed 2 years prior when he presented with splenomegaly. He commenced ruxolitinib therapy 18 months following his diagnosis when he developed progressive splenomegaly and MF‐associated symptoms. His ruxolitinib therapy was complicated by severe thrombocytopenia requiring several dose reductions, though he was able to be maintained on ruxolitinib 5 mg BD with platelets of approximately 40 to 50 × 10^9^/L. He was referred for trial due to lack of splenic response and persistent symptoms. At the time, he was deemed to be mutation and karyotype‐enhanced IPSS for primary MF version 2 (MIPSS70+ v2) very high‐risk and the spleen measured 31 cm below the costal margin. Cytogenetics were normal, however NGS revealed numerous additional mutations including ASXL1, CBL, SRSF2 and RUNX1. He has subsequently been enrolled in the PACIFICA trial (NCT03165734) and will soon be randomised and commence treatment [40].

Thrombocytopenia in MF may be disease‐associated and/or treatment‐emergent. It is a significant concern in MF patients as it is associated with a poorer prognosis (including leukaemia‐free survival) and acts as a risk factor for bleeding [2]. Thrombocytopenia represents a dose‐limiting toxicity of ruxolitinib and a reduction in platelet count is universally observed when commencing therapy. As a result, the starting dose is determined according to the platelet count. It is currently recommended that those individuals with platelets less than 50 × 10^9^/L should not receive regular ruxolitinib dosing (due to an increased risk of bleeding); however, these patients should be managed on a case‐by‐case basis with consideration of low‐dose ruxolitinib (5 mg BD) if deemed safe and appropriate. Thrombocytopenia, unlike anaemia, does not improve considerably after the first few months of therapy. However, the drop in platelet count tends to be greatest 4–12 weeks after commencing ruxolitinib, and often can effectively be managed with dose adjustments [8, 41]. Of note, thrombocytopenic MF patients still derive benefit from treatment with ruxolitinib as evidenced by the JUMP (NCT01493414) and EXPAND (NCT01317875) studies [42, 43].

There is no currently available specific therapy to improve MF‐associated thrombocytopenia. It is recommended that platelet transfusions are restricted to the appropriate contexts (i.e., platelet count less than 10 × 10^9^/L in the absence of risk factors for bleeding, or less than 20 × 10^9^/L in those with risk factors) given their limited half‐life and the propensity to develop platelet refractoriness in those that are chronically transfused. Tranexamic acid is often used as bleeding prophylaxis in these patients; however, a recent study has reported that there was no significant reduction in risk of WHO grade 2 or higher bleeding in patients with haematological malignancy and treatment‐related thrombocytopenia [44]. Corticosteroids may improve thrombocytopenia in MF, nevertheless they are not a long‐term option, and patients require close monitoring for associated side effects [45, 46, 47]. A platelet response has been observed in approximately 20% of patients treated with danazol and pomalidomide, whilst up to 81% of those receiving TGF‐β 1/3 trap, AVID200, had an increase in platelet count in recently released Phase 2 data [26, 48, 49]. This agent was associated with both spleen and symptom responses without significant toxicity, and further studies in thrombocytopenic MF setting are eagerly awaited [49].

Pacritinib is a dual JAK‐2 and fms‐related tyrosine kinase 3 (FLT‐3) inhibitor that also interacts with IRAK1 and is employed in those with baseline or treatment‐emergent thrombocytopenia. In PAC203 (NCT04884191), a Phase 2 dose‐finding study, a spleen response was evident in 17% of patients with platelets less than 50 × 10^9^/L [50]. Improvement in severe thrombocytopenia was seen in pacritinib‐treated individuals with intermediate and high‐risk MF in PERSIST‐1 (NCT01773187), whilst PERSIST‐2 (NCT02055781) showed that pacritinib was superior to best available treatment for reducing symptoms and spleen volume in those with platelet counts less than 100 × 10^9^/L [51, 52, 53]. PACIFICA (NCT03165734) is a Phase 3 trial currently recruiting severely thrombocytopenic patients with limited or no prior JAK inhibitor exposure comparing pacritinib to physician's choice of therapy. Momelitinib may also represent an alternative JAK inhibitor in this setting. Post hoc analysis of its use in randomised, Phase 3 studies have demonstrated that it is both safe and efficacious in patients with MF and moderate to severe thrombocytopenia (platelet counts < 100 × 10^9^/L) [54].

## 3 Infection

### 3.1 Herpes Zoster

#### 3.1.1 Case Study 3

A 60‐year‐old female had a diagnosis of JAK2‐positive primary MF made following the discovery of mild splenomegaly on imaging (Table 1). The initial DIPSS was 0, and she was managed on hydroxycarbamide; however, she subsequently developed constitutional symptoms with the emergence of peripheral blood blasts, so she was upgraded to a DIPSS intermediate‐2 and referred for consideration of clinical trial participation 4 years post‐diagnosis. Upon review, she was noted to have marked splenomegaly (20 cm below the costal margin) and was deemed appropriate for the REFINE trial (NCT03222609), so she commenced on ruxolitinib 10 mg BD and navitoclax [13]. Cytogenetics/FISH at the commencement of the study showed a del(6q), and the JAK2‐V617F mutation was evident at 35% VAF on NGS (along with low‐level DNMT3A and PPM1D clones). Despite requiring several navitoclax dose reductions for cytopenias, the patient had substantial improvement in both her symptoms and spleen size with this combination therapy. At Week 199 of the trial, she presented with herpes zoster involving the S1/2 dermatomes. Due to the risk of disseminated disease, she was admitted to the hospital for intravenous acyclovir, and following an improvement in her symptoms, she was de‐escalated to oral therapy. The patient has continued prophylactic valaciclovir and remains on both ruxolitinib and navitoclax; she will receive the Shingrix vaccine in the near future.

Ruxolitinib has significant immunosuppressive effects, and it has been demonstrated that the drug modifies dendritic cell function with defective T‐cell priming and hindered vaccine‐induced adenoviral clearance [10, 11, 55]. A multitude of bacterial, mycobacterial, fungal, viral and other opportunistic infections have been observed, including, but not limited to hepatitis B virus (HBV) reactivation, *Cryptococcus neoformans* pneumonia, toxoplasmosis retinitis, cytomegalovirus (CMV) retinitis, disseminated tuberculosis and progressive multifocal leucoencephalopathy [56, 57, 58, 59, 60, 61]. Consequently, it is essential to advise patients regarding the risk of developing severe infections and assess them appropriately prior to initiating therapy and throughout treatment. Screening (either serologic and/or cell‐based) should be considered for herpes simplex virus (HSV), varicella zoster virus (VZV), Ebstein–Barr virus, CMV, HBV, hepatitis C, human immunodeficiency virus (HIV), *Toxoplasma gondii* and *mycobacterium tuberculosis* (both active and latent phases) before commencing ruxolitinib [62, 63]. Some patients may require prophylactic antiviral or antibiotic therapy or additional monitoring based on their history or test results, and risk‐stratified regimes have been proposed in the past [62].

Two recent meta‐analyses of ruxolitinib clinical trials involving MPN patients have shown a higher incidence of herpes zoster infection in those treated with ruxolitinib [64, 65]. Patients should therefore be informed regarding the signs and symptoms of herpes zoster so that they can seek medical intervention as early as possible. Moreover, a non‐live recombinant adjuvant zoster vaccine (Shingrix) is now available for protection against herpes zoster infection and herpetic neuralgia and has been shown to be highly effective in the immunocompromised population (up to 91%). It is therefore recommended that patients with MF undergo vaccination with Shingrix when possible. It is unclear whether valaciclovir prophylaxis is required following Shingrix; however, it might be reasonable to initiate long‐term prophylaxis in those that develop herpes zoster following the vaccine.

## 4 Secondary Malignancy

### 4.1 Non‐Melanomatous Skin Cancers

#### 4.1.1 Case Study 4

A 74‐year‐old male commenced ruxolitinib 10 mg BD after being diagnosed with post‐ET MF (DIPSS high‐risk) (Table 1). Prior to the transformation of his JAK2‐positive ET, he had received hydroxycarbamide for 9 years. His past history included multiple skin cancers (squamous cell carcinoma [SCC], basal cell carcinoma [BCC] and melanoma). The ruxolitinib dose was increased to 20 mg BD over 6 months to attempt to gain better control of the disease, and this resulted in some reduction in splenic volume and MPN‐associated symptoms. The patient was interested in the possibility of disease modification, and he subsequently received an MDM2 inhibitor (siramadlin) with ruxolitinib as part of the ADORE study (NCT04097821) [66]. He developed multiple SCCs and BCCs during the trial, requiring surgical resection. Siramadlin was ceased after eight cycles due to cytopenias and a lack of splenic and symptomatic improvement; however, ruxolitinib was continued at a lower dose (10 mg BD), along with frequent dermatological assessments. Several months later, the patient had navitoclax added to his therapy on the REFINE clinical trial (NCT03222609), which resulted in a decrease in his spleen volume [13]. After 12 months on the trial, he developed cervical lymphadenopathy, and investigations revealed metastatic Merkel cell carcinoma arising from a primary scalp lesion. He received radiotherapy with a good response. He has had further SCCs treated with acitretin, cryotherapy and surgery. Ruxolitinib therapy has been continued throughout, and his MF remains stable.

Non‐melanomatous skin cancers (NMSC), which include basal cell, squamous cell and Merkel cell carcinoma, have been reported in patients that have received ruxolitinib; however, a clear causal relationship has yet to be established (but is highly likely). Most patients that develop NMSC have had prolonged treatment with hydroxycarbamide and a history of NMSCs and/or pre‐malignant skin lesions. The rates of BCC and SCC were not significantly different between treatment groups in COMFORT‐1 (NCT00952289); however, a higher incidence was noted in patients assigned to ruxolitinib in the COMFORT‐2 study (NCT00934544), and a number of reports have emerged since in support of this association [67, 68, 69, 70, 71, 72]. The incidence of SCC might be higher in continents with high lifetime UV exposure, such as Australia and Africa. Given this, it is recommended that individuals on ruxolitinib have periodic skin examinations and advice about skin protection and that those diagnosed with NMSC have early intervention to optimise treatment outcomes [63]. The decision to continue or cease therapy is a difficult one. The risk of NMSC is likely to be present with other JAK inhibitors (a class effect), so changing to an alternative JAK inhibitor, if available, may not reduce the risk; though, ultimately, longitudinal safety data from studies using other JAK inhibitors is needed to determine this.

## 5 Progressive Disease

### 5.1 Worsening MF and Ruxolitinib Suboptimal Response/Refractoriness

#### 5.1.1 Case Study 5

A 61‐year‐old male was diagnosed with JAK2‐positive primary MF (Table 1). He commenced ruxolitinib 12 years post‐diagnosis for symptomatic disease however; this was ceased due to progressive anaemia. He was subsequently enrolled in the BOREAS trial (NCT03662126), where he received an MDM2 inhibitor (navtemadlin), though he did not continue beyond cycle six due to a lack of response and worsening symptoms [73]. During the study, he developed significant upper and lower gastrointestinal bleeding and was found to have oesophageal varices, portal hypertensive gastritis and haemorrhoids. He also experienced fluid overload, including intermittent ascites, requiring treatment with diuretics. He was evaluated extensively for the cause of his portal hypertension, and it was concluded that this was secondary to liver involvement by his MF. There was no portal or hepatic vein thrombosis. The patient was recommenced on ruxolitinib, as well as hydroxycarbamide, which resulted in a reduction in his spleen size and symptoms, although he became transfusion dependent. He is now on the FREEDOM‐2 trial (NCT03952039) receiving fedratinib, which has improved his symptoms, splenomegaly and transfusion requirements [74]. His liver disease has been managed by the gastroenterologists using a combination of low‐dose diuretics and regular paracentesis.

#### 5.1.2 Case Study 6

A 74‐year‐old male with a 20‐year history of JAK2‐positive ET was diagnosed with post‐ET MF (Table 1). He started ruxolitinib at 10 mg BD and had an excellent spleen response (from over 16 cm below the costal margin to not palpable) but developed transfusion dependency. After 3 years, he began to lose response, developing new splenomegaly (17 cm below the costal margin), progressive MPN‐related symptoms, and an increasing white cell count (18 × 10^9^/L). A bone marrow biopsy showed no evidence of BP disease; however, NGS demonstrated the acquisition of an ASXL1 mutation. The dose of ruxolitinib was increased to 15 mg BD without effect, and he was therefore weaned from ruxolitinib to begin fedratinib (as part of the FREEDOM‐2 trial [NCT03952039]; yet this had to be abandoned due to the development of progressive hepatosplenomegaly and symptom recurrence (presumably due to ruxolitinib discontinuation) [74]. The patient was recommenced on ruxolitinib 10 mg BD with the addition of navitoclax via the REFINE study [13]. After almost 6 months of study, his disease continued to progress with worsening splenomegaly (24 cm below the costal margin) and increasing leucocytosis (26 × 10^9^/L) prompting the cessation of navitoclax. The dose of ruxolitinib was increased to 15 mg BD, and hydroxycarbamide was initiated. His spleen size decreased (11 cm below the costal margin), and symptoms improved somewhat initially; however, over the next 2 years, his white cell count continued to increase (up to 265 × 10^9^/L), and he developed thrombocytopenia requiring close titration of his therapy. The addition of pegylated interferon was also trialled but had no effect. He presented acutely unwell with a white cell count of 422 × 10^9^/L (3% peripheral blood blasts) and received palliative care due to an inability to tolerate more intensive therapy. He died within 24 h of presentation.

There are no well‐defined criteria for ruxolitinib failure warranting a change in therapy. Many patients may still be benefitting from ruxolitinib even as they are beginning to lose their response. The International Working Group on Myeloproliferative Neoplasms Research and Treatment (IWGMRT) criteria are used for response assessment and can help define progression or poor response, particularly in the clinical trial context [38]. Nevertheless, they tend to focus on splenomegaly and leukaemic transformation and do not take into account other manifestations of progression (such as increasing anaemia, thrombocytopenia, leucocytosis, symptoms or extramedullary haematopoiesis), nor does it incorporate ruxolitinib dose [75]. It has been proposed that alternative treatments should be pursued in the context of worsening symptoms and cytopenias/cytoses, or increasing blast count and splenomegaly (including persistence of significant splenomegaly [> 10 cm from the costal margin] despite having decreased 50% or more initially) [76]. It is also worth noting that the prognosis for patients that have clonal evolution whilst on ruxolitinib is especially poor and can herald progression [77].

The Canadian Myeloproliferative Neoplasm Group refers to ruxolitinib failure as being either resistance or intolerance to the drug, and that it frequently presents as ‘loss or failure to obtain a significant reduction in splenomegaly or symptom response’ and ‘the development or persistence of clinically significant cytopenia’ [78]. Ruxolitinib dose modification may improve response in some instances, whilst in others, alternative therapies should be pursued.

JAK inhibition with an alternative agent is a logical approach in patients who are refractory to ruxolitinib. Fedratinib is a pan‐JAK inhibitor that has established efficacy in the setting of ruxolitinib failure [79]. In the JAKARTA‐2 study (NCT01523171), spleen and symptom response were 30% and 27%, respectively, with a 1‐year survival of 84% [80, 81]. The FREEDOM‐2 trial (NCT03952039) has evaluated fedratinib in comparison to the best available therapy and shown superior spleen and symptom response rates following six cycles of treatment, at 35.8% versus 6.0% and 34.1% versus 16.9%, respectively [74]. As fedratinib is associated with Wernicke's encephalopathy, it is essential that thiamine testing is undertaken when initiating therapy (with replacement as necessary and prophylactic supplementation). In patients with suboptimal responses or haematologic adverse effects with ruxolitinib, momelotinib leads to greater symptomatic improvement and a better anaemia response when compared to the best available therapy; however, it was not shown to be superior for reduction in spleen size, whilst pacritinib is as an option in those patients with thrombocytopenia [34].

Hydroxycarbamide can also help in alleviating splenomegaly, cytoses and constitutional symptoms (provided there is no significant pre‐existing anaemia) [82, 83].

Numerous other compounds (including BCL‐2, PI3Kδ, telomerase, BET, MDM2 and XPO1 inhibitors) are being explored in the trial setting both as a monotherapy and in combination with ruxolitinib. Navitoclax has shown promising results in the REFINE study (NCT03222609), where it was combined with ongoing ruxolitinib therapy in patients with MF who had progression or suboptimal response. 41% of the cohort achieved a spleen response, and symptomatic improvement was reported in 30% [13]. Such a combination offers potential for disease modification with improvement in bone marrow fibrosis seen in a third of patients and evidence of a molecular response (VAF reductions of 20% or more in up to 28% of the group); both correlated positively with survival [13, 84]. In BOREAS (NCT03662126), the MDM2 inhibitor, navtemadlin, also demonstrated bone marrow fibrosis improvement and a reduction in VAFs of driver mutations, with spleen and symptom response [85, 86]. Despite such effects, a survival advantage has yet to be seen [86]. The MANIFEST trial (NCT02158858) investigated the BET inhibitor, pelabresib, both as a mono‐ and combination therapy, in a number of different settings, including those who were refractory or had suboptimal or lost response to ruxolitinib. Improvements in transfusion dependence, haemoglobin levels and bone marrow fibrosis were seen in this patient group [87, 88].

### 5.2 Accelerated and BP

#### 5.2.1 Case Study 7

A 72‐year‐old man was diagnosed with JAK2‐positive PMF (Table 1). He had normal cytogenetics and an ASXL1 mutation on NGS; he was classified as intermediate‐2 disease according to DIPSS Plus. Ruxolitinib was initiated, and there was some improvement in his disease. After a year on treatment, he was noted to have progressive splenomegaly, worsening symptoms and developed a marked leucocytosis (111 × 10^9^/L) and anaemia (despite ESA therapy). He was referred for a clinical trial and participated in the ADORE study (NCT04097821), receiving ERK inhibitor, rineterkib, in addition to 20 mg of ruxolitinib BD [66]. Despite this, there was no significant change in the patient's disease status, and he developed retinopathy, necessitating withdrawal from this clinical trial. He then commenced TRANSFORM‐2 (NCT04468984), receiving ruxolitinib plus navitoclax [89]. His navitoclax was withheld from Week 3 of the trial due to thrombocytopenia, and then within a fortnight, he developed progressive disease with an increase in his white cell count from 144 to 217 × 10^9^/L (with 1% peripheral blood blasts) and persistent thrombocytopenia, precluding further participation in this trial. He was managed with the combination of ruxolitinib and hydroxycarbamide for 8 months, until he transformed to BP disease (46% peripheral blood blasts). He commenced azacitidine and venetoclax, and a bone marrow after the first cycle demonstrated a hypocellular marrow with no definite increase in blasts. He received a second cycle; however, this was complicated by pancytopenia, massive symptomatic splenomegaly and pneumonia. A subsequent bone marrow biopsy was profoundly hypocellular. He was given supportive care, including 4 Gy splenic radiotherapy and managed palliatively within the community. Over a 10‐day period, his white cell count rose rapidly to 265 × 10^9^/L; at this point, no further therapy was thought to be appropriate, and he died several days later.

Progression to BP of the disease is defined by 20% or more circulating or bone marrow blasts. It is typically proceeded by a period of lower‐level excess blasts (10%–19% in blood or bone marrow), which is referred to as the accelerated phase (AP) of MF. This may be heralded by worsening leucocytosis, cytopenias (particularly the development of thrombocytopenia on a stable dose of ruxolitinib), splenomegaly and/or constitutional symptoms. Transformation to acute myeloid leukaemia (AML) is seen in 10%–15% of MF cases overall and carries a dismal prognosis [90, 91, 92].

Both the DIPSS and the MIPSS70+ v2 can be employed to predict likelihood of evolution to BP with risk factors for transformation including age over 70 years, moderate to severe anaemia, thrombocytopenia, circulating blasts of 3% or more, high‐risk/unfavourable karyotype and *IDH1*, *IDH2*, *SRSF2* or *ASXL* mutations [91, 92, 93, 94, 95, 96]. Other factors associated with poorer leuakaemia‐free survival include transfusion‐requiring anaemia, elevated serum interleukin‐9 and C‐reactive protein, and several genetic factors including monosomal karyotype, triple‐negative disease and, *RUNX1*, *CEBPA*, *SH2B3* and RAS/MAPK pathway gene variants [97].

No standard treatment for transformation to BP disease exists and clinical trial enrolment, where possible, is recommended. AHSCT should be explored in appropriate patients ideally prior to the development of BP disease as this is the only curative therapy in this context and is associated with 3‐year survival or more than 30% [98]. Intensive chemotherapy (with AML induction‐like regimes) without consolidative AHSCT does not lead to a durable response [99, 100, 101]. In patients unfit for AHSCT, treatment options include lower intensity therapy and supportive care. Regimes incorporating hypomethylating agents, BCL‐2 inhibitors (e.g., venetoclax), IDH inhibitors and ruxolitinib have been used with varying response rates, but typically have poor median overall survival with durations less than 12 months [98, 102–109]. Ultimately, there remains an urgent unmet need for more effective interventions in this patient population.

## 6 Conclusions

The treatment landscape in MF is evolving rapidly, however ruxolitinib remains the standard of care and has been established as a safe and effective compound in clinical trials, pooled analyses, expanded‐access studies and post marketing surveillance [110]. Haematological adverse events are expected with JAK inhibition and can be managed with dose modification, transfusion, or a combination of the two. Whilst careful monitoring and prompt intervention for non‐haematological side effects, such as infection and secondary malignancy, is essential with any immunosuppressive therapy. A range of therapies are being explored in the context of ruxolitinib failure, whilst some of the novel agents (including as a dual treatment with ruxolitinib) offer potential for disease modification (Table 1).

## Conflicts of Interest

L.A.B. received speaker fees from Novartis and travel funding to meeting from Amgen, BMS and Novartis. C.F. received speaker fees from Novartis and served as advisory role for GSK and Novartis. C.H. received research funding from GSK, Morphosys and Novartis; served as advisory role for AbbVie, AOP, BMS, CTI, Galacteo, Geron, GSK, Imago, Incyte, Ionis, Janssen, Keros, Morphosys, MSD, Novartis, Silence and SOBI; received speaker fees from BMS, CTI, GSK, Incyte and Novartis; and travel funding to meeting from Novartis. A.C.P. served as advisory role for CTI, GSK, Kartos Therapeutics, Novartis, Silence Therapeutics and Incyte.

## Clinical Trial Registration

The authors have confirmed clinical trial registration is not needed for this submission.

## Data Availability

Data available upon request due to privacy/ethical reasons
